# Detection of early ocular circulatory changes in Takayasu arteritis using laser speckle flowgraphy

**DOI:** 10.1016/j.ajoc.2026.102574

**Published:** 2026-03-27

**Authors:** Kensuke Mimura, Yoichiro Shinohara, Hideo Akiyama

**Affiliations:** Department of Ophthalmology, Gunma University Graduate School of Medicine, 3-39-15 Showa-machi, Maebashi, Gunma, 371-8511, Japan

**Keywords:** Takayasu arteritis, Laser speckle flowgraphy, Ocular blood flow, Steroid therapy, Optical coherence tomography angiography

## Abstract

**Purpose:**

To describe a case of early-stage Takayasu arteritis (TA) in which ocular hypoperfusion was detected using laser speckle flowgraphy (LSFG) and subsequently monitored during systemic corticosteroid therapy.

**Observations:**

A 48-year-old man presented with transient bilateral blurred vision during exercise. The initial ophthalmic examination revealed structural abnormalities on optical coherence tomography, including thinning of the retinal ganglion cell-inner plexiform layer. LSFG showed reduced blood flow in the optic nerve head and choroidal regions, while optical coherence tomography angiography (OCTA) demonstrated decreased retinal vascular density. He was diagnosed with TA based on systemic inflammation, hypotension, and positron emission tomography/computed tomography findings of large-vessel involvement. High-dose oral corticosteroid therapy normalized blood pressure and inflammatory markers, accompanied by a rapid improvement in LSFG-derived ocular perfusion and increased retinal vascular density on OCTA.

**Conclusion:**

This case illustrates that ocular hypoperfusion may be present in early-stage TA even in the absence of advanced fundus features of Takayasu retinopathy. LSFG can detect dynamic and reversible perfusion abnormalities, and when combined with OCTA, provides a sensitive, noninvasive approach for identifying early ocular involvement and monitoring treatment response in TA.

## 1 Introduction

Takayasu arteritis (TA) was first described in 1908 by Japanese ophthalmologist Mikito Takayasu, who reported characteristic changes in the central retinal vessels. It is now recognized as a rare, chronic granulomatous vasculitis predominantly affecting the aorta and its major branches.^1^^,^^2^ TA primarily affects young women under 40 years of age, with a male-to-female ratio of up to 1:9 and an annual incidence of 1–2 per million. The disease is most prevalent in Asia and South America.^3^ Ocular involvement, reported in up to 68% of patients, includes Takayasu retinopathy, optic neuropathy, ocular ischemic syndrome, and retinal vascular occlusion, all resulting from reduced perfusion due to carotid or ophthalmic artery stenosis.^4^

Most previous reports have focused on ocular manifestations in the chronic phase of TA. In early stages, fundus findings may be subtle or absent, highlighting the need for sensitive diagnostic tools. Conventional imaging modalities, including fluorescein angiography (FA), indocyanine green angiography, and optical coherence tomography angiography (OCTA), provide valuable structural and vascular information but may fail to capture dynamic hemodynamic changes in early disease.^5^^,^^6^ For example, retinal arteriovenous shunts on FA are typically observed only in advanced disease.^7^

Laser speckle flowgraphy (LSFG) is an emerging, noninvasive technique that quantifies blood flow through the mean blur rate (MBR), reflecting erythrocyte movement. LSFG demonstrates high reproducibility and utility in assessing the optic nerve head (ONH) and choroidal circulation in various ocular disorders.^8^ However, its application in systemic vasculitis such as TA has not been previously reported. Here, we present a case in which LSFG was used to assess ocular blood flow changes in TA before and after systemic steroid therapy, demonstrating its potential for detecting early hemodynamic alterations prior to the development of overt late-stage vascular complications.

## 2 Case presentation

**Fig. 1:**
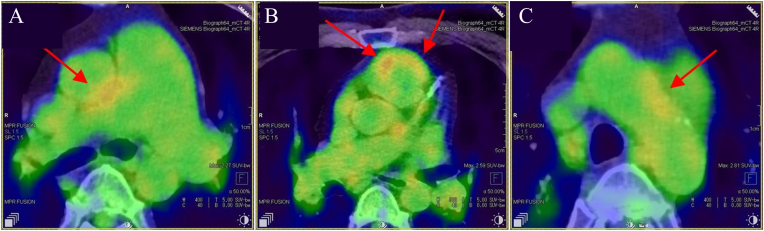
Baseline FDG-PET showing systemic vascular inflammation in Takayasu arteritis. Red arrows indicate ^18F-fluorodeoxyglucose uptake along the ascending aorta (SUVmax, 4.0; A), main pulmonary artery (SUVmax, 4.1; B), and proximal right subclavian artery (SUVmax, 3.4; C), consistent with active vascular inflammation. (For interpretation of the references to color in this figure legend, the reader is referred to the Web version of this article.)

A 48-year-old man was referred to our department with transient bilateral blurred vision during physical exertion. He had a history of atopic dermatitis and had been diagnosed with subacute myocardial infarction and pulmonary hypertension 3 months earlier. At the initial visit, his blood pressure was 76/48 mmHg. Laboratory tests showed leukocytosis (11,300/μL), elevated C-reactive protein (2.59 mg/dL), and an increased erythrocyte sedimentation rate (ESR) (48 mm/h). Positron emission tomography–computed tomography (PET-CT) revealed increased ^18F-fluorodeoxyglucose uptake in the ascending aorta (SUVmax, 4.0), main pulmonary artery (SUVmax, 4.1), and proximal right subclavian artery (SUVmax, 3.4), consistent with active vascular inflammation (Fig. 1A–C). TA was diagnosed based on the 2022 American College of Rheumatology classification criteria.^9^

**Fig. 2:**
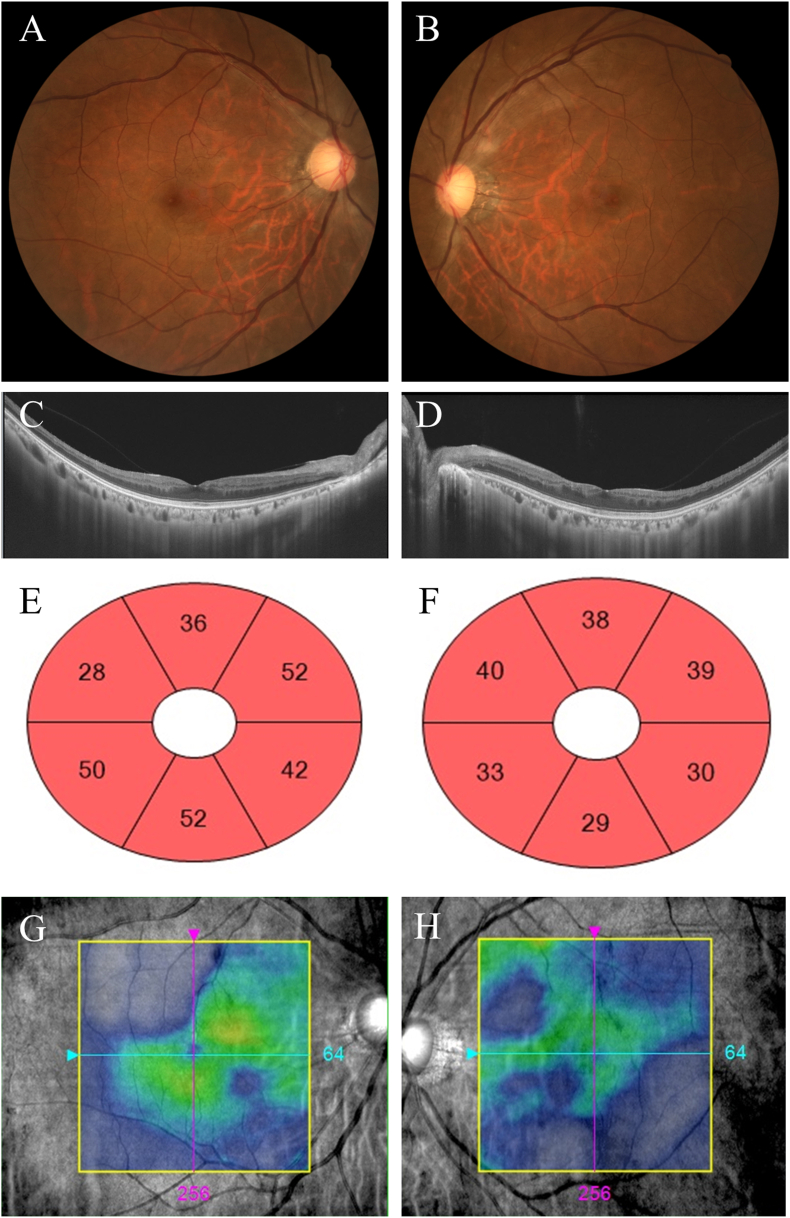
Baseline fundus and optical coherence tomography (OCT) images illustrating the overall retinal appearance, with OCT demonstrating areas of partial retinal thinning. (A, B) Color fundus photographs of the right and left eyes did not show features of advanced TA. (C, D) Swept-source OCT B-scans of the right and left eyes showed a slightly shallow fovea. (E, F) Ganglion cell-inner plexiform layer (GCIPL) sectors maps of right and left eyes showed reduced macular GCIPL thickness. (G, H) Cirrus high-definition OCT of both eyes demonstrated diffuse full-thickness retinal thinning from the inner limiting membrane to the retinal pigment epithelium. (For interpretation of the references to color in this figure legend, the reader is referred to the Web version of this article.)

Ophthalmologic examination revealed best-corrected visual acuity of 20/16 in both eyes and intraocular pressure of 10 mmHg bilaterally. Slit-lamp examination was unremarkable. Color fundus photographs did not show features of advanced Takayasu retinopathy such as microaneurysms, arteriovenous shunts, or hemorrhage (Fig. 2A and B). Swept-source optical coherence tomography (SS-OCT; DRI Triton; Topcon, Tokyo, Japan) revealed slightly shallow fovea compared with those of normal eye (Fig. 2C and D), whereas Cirrus high-definition OCT (Cirrus HD-OCT; Carl Zeiss Meditec, Dublin, CA, USA) showed thinning of the ganglion cells and inner plexiform layer (GCIPL) in both eyes (Fig. 2E and F), along with diffuse full-thickness retinal thinning from the inner limiting membrane to the retinal pigment epithelium (Fig. 2G and H). These findings may be compatible with chronic retinal hypoperfusion.

**Fig. 3:**
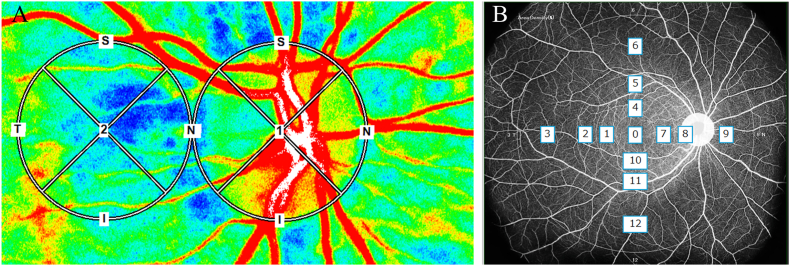
Methods for laser speckle flowgraphy (LSFG) and optical coherence tomography angiography (OCTA) analysis of ocular blood flow. (A) LSFG images were obtained using the LSFG-NAVI system. For analysis, mean blur rate (MBR) images of the optic nerve head (ONH) and choroidal regions were acquired. Based on previous reports, a circular region of interest (ROI) was placed around the ONH, followed by an identical ROI positioned laterally to delineate the choroidal region. Blood flow at the ONH was assessed using vessel-area MBR and tissue-area MBR. To evaluate choroidal blood flow, an ROI of the same size as the optic disc was set adjacent to it. (B) Wide-field OCTA (Xephilio OCT-S1) showing 13 sectors: central fovea (area 0) and 12 surrounding sectors. A 2.5-mm peripapillary region centered on the ONH was excluded. Sector numbering started temporally from the innermost ring in a clockwise direction (temporal–superior–nasal–inferior).

**Fig. 4:**
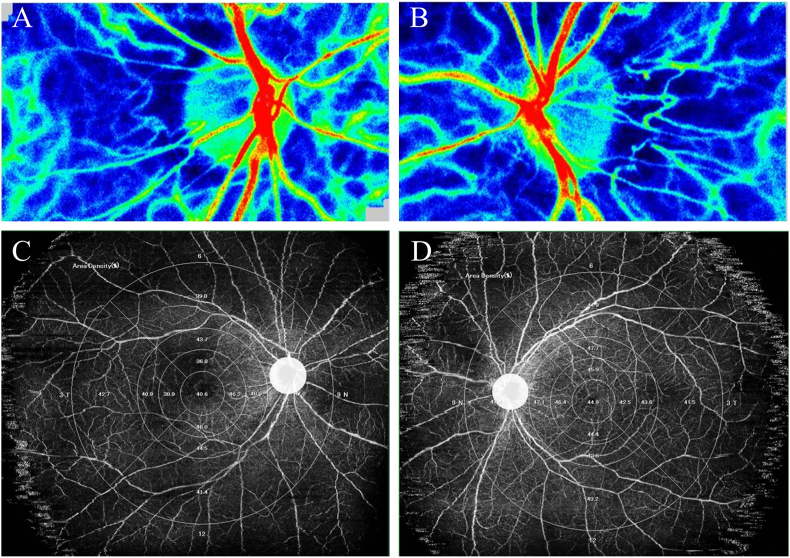
Baseline LSFG and OCTA (superficial retinal capillary plexus) revealing early ocular blood flow reduction. (A, B) LSFG color maps of the right and left eyes showing reduced ocular blood flow relative to reported normative values.^12^ (C, D) OCTA images of both eyes showing generalized retinal vascular density reduction with localized capillary rarefaction. (For interpretation of the references to color in this figure legend, the reader is referred to the Web version of this article.)

Ocular blood flow was evaluated using LSFG (LSFG-NAVI; Softcare Co., Ltd., Fukuoka, Japan). The MBRs of the ONH and choroidal regions were determined. A circular region of interest (ROI) was manually drawn around the ONH to assess blood flow, and an identical ROI was placed laterally to delineate the choroidal region, as previously described^10^ (Fig. 3A). Blood flow in the ONH was assessed using the vessel-area MBR (MV) and tissue-area MBR (MT) components. All LSFG examinations were conducted under standardized conditions between 10:00 a.m. and 3:00 p.m. after at least 10 minutes of seated rest and under mydriasis induced with Mydrin-P (0.5% tropicamide and 0.5% phenylephrine hydrochloride). OCTA was performed using a wide-field SS-OCT system (Xephilio OCT-S1; Canon Medical Systems, Tokyo, Japan). Montaged scans covering 23 × 20 mm were analyzed using the OCT Research Tool version 3.0 (Canon Inc., Tokyo, Japan).^11^ The central foveal region was designated as Area 0, and the surrounding retina was divided into 12 equidistant sectors based on concentric circles with diameters of 3, 6, 9, and 18 mm, resulting in 13 sectors. A 2.5-mm circular peripapillary region centered on the ONH was excluded from analysis. Sector numbering began temporally from the innermost ring and proceeded outward in a clockwise manner (temporal-superior-nasal-inferior), ensuring anatomical symmetry between the right and left eyes (Fig. 3B). Baseline LSFG showed reduced ocular blood flow in both eyes (Fig. 4A and B). ONH-MBR values were 36.4 (MV) and 8.4 (MT) in the right eye and 27.2 (MV) and 7.1 (MT) in the left eye, which were lower than the reported normative means (MV, 42.6 ± 6.6; MT, 12.8 ± 2.8).^12^ Baseline OCTA image of the superficial retinal capillary plexus revealed a generalized reduction in retinal vascular density, more pronounced in the right eye, with localized areas of marked capillary rarefaction within the vascular arcade, without enlargement of the foveal avascular zone (FAZ) (Fig. 4C and D).

**Fig. 5:**
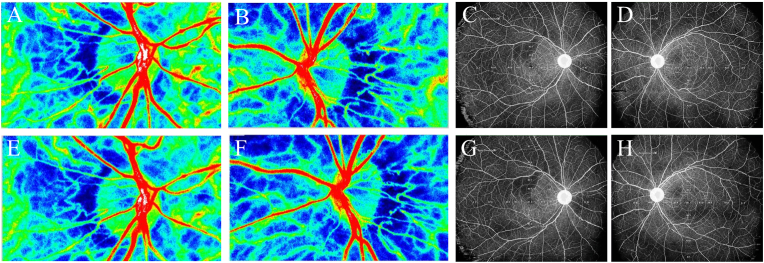
LSFG and OCTA demonstrating ocular blood flow recovery after systemic corticosteroid therapy. (A, B) LSFG color maps at 1 month showing marked improvement in ocular blood flow. (C, D) OCTA images at 1 month showing increased retinal vascular density compared with baseline. (E, F) LSFG at 4 months showing sustained blood flow improvement. (G, H) OCTA at 4 months demonstrating continued recovery of retinal vascular density. (For interpretation of the references to color in this figure legend, the reader is referred to the Web version of this article.)

High-dose oral prednisolone (PSL; 50 mg/day) was initiated. Two weeks later, blood pressure improved to 117/87 mmHg, and inflammatory markers normalized (WBC 5000/μL, CRP 0.05 mg/dL, ESR 10 mm/h). One month after treatment, when the PSL dose had been tapered to 30 mg/day, LSFG showed marked improvement in ocular blood flow (Fig. 5A and B), and OCTA demonstrated increased retinal vascular density (Fig. 5C and D). At 4 months, with PSL further reduced to 15 mg/day, systemic and ocular parameters remained stable, with sustained LSFG and OCTA improvements (Fig. 5E–H). Corticosteroids were gradually tapered without recurrence. Central retinal thickness (CRT) was 220 μm at baseline and 223 μm at 4 months in the right eye and 239 μm at baseline and 227 μm at 4 months in the left eye. Central choroidal thickness (CCT) was 154 μm at baseline and 215 μm at 4 months in the right eye and 134 μm at baseline and 154 μm at 4 months in the left eye. No appreciable change in GCIPL thickness was observed during follow-up. The longitudinal changes in blood pressure, LSFG, and retinal vascular density are summarized in Table 1, Table 2.

**Table 1:** Changes in blood pressure and ocular blood flow measured by laser speckle flowgraphy before and after treatment.

	sBP (mmHg)	dBP (mmHg)	PSL (mg/day)	Right eye			Left eye		
				ONH-MV	ONH-MT	CHOR-MBR	ONH-MV	ONH-MT	CHOR-MBR
initial visit	76	48	0	36.4	8.4	3.3	27.2	7.1	3.5
1 month	125	83	30	48.4 (+33%)	10.3 (+23%)	5.4 (+64%)	32.0 (+18%)	8.4 (+18%)	5.6 (+60%)
4 months	120	80	15	48.7 (+34%)	10.2 (+21%)	4.9 (+49%)	29.4 (+8%)	7.0 (−1%)	4.8 (+37%)

sBP, systolic blood pressure; dBP, diastolic blood pressure; PSL, prednisolone; ONH, optic nerve head; MBR, mean blur rate; ONH-MV, ONH vessel-area MBR; ONH-MT, ONH tissue-area MBR; CHOR-MBR, choroidal MBR.

Percentage change was calculated relative to baseline (initial visit).

**Table 2:** Changes in retinal vascular density measured by optical coherence tomography angiography before and after treatment.

	eye	Area												
		0	1	2	3	4	5	6	7	8	9	10	11	12
initial visit	R	40.6	38.9	40.9	42.7	36.8	43.7	39.8	46.2	48.0	47.0	46.0	44.5	41.4
1 month	R	46.6	44.8	43.4	42.6	43.9	42.7	45.0	48.8	48.9	48.7	46.4	46.3	44.3
4 months	R	47.1	45.5	45.3	45.2	46.4	44.6	45.5	48.5	49.3	47.7	47.6	45.9	44.9

initial visit	L	44.9	42.5	43.6	41.5	45.9	47.7	44.9	46.4	47.1	47.4	44.4	43.8	43.2
1 month	L	46.6	46.4	46.0	44.1	47.4	47.8	45.3	47.0	48.3	49.8	46.2	45.8	43.4
4 months	L	45.7	43.8	44.9	42.6	46.9	48.0	47.2	45.4	48.7	48.6	43.1	44.9	43.8

R, right; L, left.

## 3 Discussion

This case demonstrates, using LSFG, that TA can cause ocular ischemia before advanced fundus features of Takayasu retinopathy appear. LSFG revealed markedly reduced blood flow in both the ONH and choroidal regions at the initial visit and subsequently captured a rapid restoration of ocular perfusion following systemic corticosteroid therapy. These findings highlight the utility of LSFG in identifying early hemodynamic alterations and in monitoring treatment response before overt retinal vascular tortuosity, dilation, or hemorrhage appears.

Previous studies have focused on advanced ocular lesions of TA, with Takayasu retinopathy characterized by microaneurysms, arteriovenous shunts, and retinal vascular occlusions‒typically identified using fundus photography and FA. However, even when the retina appears without obvious abnormalities, TA patients may exhibit subclinical ocular circulatory changes such as elevated resistive indices in the ophthalmic and central retinal arteries.^13^ In our case, although fundus photography did not show features of advanced Takayasu retinopathy, Cirrus HD-OCT revealed areas of retinal thinning. These structural changes are consistent with chronic ocular hypoperfusion.

Noninvasive evaluation of ocular blood flow is crucial for the early detection of ischemia in TA. LSFG has previously been validated as a reliable tool for assessing ocular circulation in macrovascular disorders such as internal carotid artery stenosis^14^ and internal carotid–cavernous sinus fistula (CCF).^15^ Further analyses of CCF have shown that LSFG can capture dynamic vascular changes before and after treatment.^16^ Similar observations have been reported in inflammatory ocular diseases such as Vogt–Koyanagi–Harada disease, in which LSFG detects treatment-associated changes in ONH and choroidal circulation during systemic corticosteroid therapy^17^ .^18^ These findings support the utility of LSFG for evaluating ocular hemodynamics in conditions affecting ocular perfusion. In this case, LSFG detected early ocular hypoperfusion before ophthalmoscopic abnormalities appeared and enabled close monitoring of the rapid hemodynamic response to systemic corticosteroid therapy. Systemic treatment restored ocular perfusion, likely through anti-inflammatory effects and subsequent relief of arterial stenosis. These results align with findings in arteritic anterior ischemic optic neuropathy, where LSFG showed marked baseline hypoperfusion with significant improvement after corticosteroid therapy, supporting its role in monitoring treatment response in inflammatory vasculopathies.^19^ LSFG-derived MBR has shown high reproducibility, with coefficients of variation of 2.9% in the ONH and 4.1% in the choroid.^8^ In this case, the magnitude of MBR increase substantially exceeded these variability levels. For example, ONH-MV increased by up to 34% and choroidal MBR by up to 64% in the right eye, indicating that the observed changes were unlikely to reflect measurement fluctuation alone.

The observed increase in MBR and retinal vascular density after treatment is likely multifactorial. In TA, systemic corticosteroid therapy reduces vascular inflammation and may improve arterial stenosis, thereby enhancing blood flow. Systemic blood pressure also improved after treatment and remained stable during follow-up, which may have contributed to increased ocular perfusion pressure. Corticosteroids have also been reported to increase ONH blood flow even in unaffected fellow eyes of patients with unilateral optic neuritis, suggesting that a direct pharmacologic effect on ocular circulation cannot be excluded.^20^ In this case, however, perfusion improvement persisted after steroid tapering during follow-up. This sustained response is more consistent with improvement in arterial stenosis and systemic hemodynamics than with a transient local steroid effect. CCT slightly increased during follow-up, whereas CRT and GCIPL thickness remained largely unchanged. The increase in CCT may reflect hemodynamic alterations associated with improved ocular perfusion, although its clinical significance remains uncertain.

Previous OCTA studies in TA have mainly focused on quantitative analysis of the FAZ, reporting its enlargement in affected patients.^4^^,^^6^ OCTA has also been used to visualize optic disc neovascularization in advanced stages of Takayasu retinopathy.^21^ However, few studies have assessed retinal capillary structures using wide-field OCTA in early-stage TA. In the present case, OCTA revealed a localized reduction in retinal vascular density, while LSFG demonstrated a concurrent decrease in the ocular blood flow. Notably, both parameters improved after systemic corticosteroid therapy, in parallel with normalization of inflammatory markers and blood pressure. These findings suggest that early ocular ischemia in TA is potentially reversible with timely treatment.

The complementary roles of LSFG and OCTA are evident in detecting and monitoring early ocular involvement in systemic vasculitis. In particular, LSFG serves not only as a sensitive tool for identifying subclinical ischemia but also as a functional biomarker for evaluating treatment efficacy in TA. Structural assessment also provides important information for interpreting ocular perfusion findings. The subtle retinal thinning observed on OCT may reflect chronic ocular hypoperfusion, although evidence supporting a direct causal relationship between ischemia and such structural alterations in early-stage TA remains limited. In this case, quantitative analysis of retinal structural parameters, including retinal ganglion cell layer thickness, showed no significant improvement during follow-up despite recovery of ocular blood flow. This dissociation between functional improvement and persistent structural thinning suggests that once structural damage is established, it may not be reversible even if perfusion is restored. In some patients with TA, medical therapy alone may not sufficiently restore arterial perfusion, and surgical revascularization may be required to improve blood flow.^4^ Persistent ocular hypoperfusion may contribute to progressive retinal structural damage and potentially irreversible visual impairment. Accordingly, LSFG-derived perfusion parameters may serve as useful biomarkers for evaluating hemodynamic response to medical therapy. Limited or absent improvement in ocular blood flow could indicate insufficient restoration of arterial circulation and help identify patients who may benefit from surgical intervention.

Further studies with larger patient cohorts are warranted to validate these observations. Expanding this approach could clarify the temporal relationship between systemic inflammation, ocular perfusion, and retinal structural changes, supporting early intervention strategies aimed at preserving visual function in TA.

This report has several limitations. The patient had a history of atopic dermatitis, subacute myocardial infarction, and pulmonary hypertension, which may have influenced baseline ocular perfusion and retinal structural parameters. Systemic cardiovascular conditions are known to affect ocular blood flow,^22^ and their contribution to the observed hemodynamic findings cannot be completely excluded. Therefore, the extent to which baseline ocular hypoperfusion and structural changes were attributable solely to TA remains uncertain.

## 4 Conclusion

Early-stage TA may be associated with ocular hypoperfusion even in the absence of advanced fundus features of Takayasu retinopathy. Our report demonstrated early ocular ischemia detected by LSFG with rapid restoration of blood flow following systemic corticosteroid therapy. These findings suggest that LSFG, particularly when combined with OCTA, may serve as a valuable noninvasive tool for detecting early ocular involvement and monitoring therapeutic response in TA.

## CRediT authorship contribution statement

**Kensuke Mimura:** Writing – original draft, Investigation, Formal analysis, Data curation. **Yoichiro Shinohara:** Writing – review & editing, Writing – original draft, Visualization, Validation, Supervision, Project administration, Methodology, Investigation, Data curation, Conceptualization. **Hideo Akiyama:** Writing – review & editing, Visualization, Supervision, Investigation, Conceptualization.

## Patient consent

Written informed consent was obtained from the patient for publication. This report does not contain any personal identifying information.

## Authorship

All authors attest that they meet the current ICMJE criteria for Authorship.

## Funding

No funding or grant support.

## Declaration of competing interest

The authors declare that they have no known competing financial interests or personal relationships that could have appeared to influence the work reported in this paper.
